# EphA2 Is a Therapy Target in EphA2-Positive Leukemias but Is Not Essential for Normal Hematopoiesis or Leukemia

**DOI:** 10.1371/journal.pone.0130692

**Published:** 2015-06-17

**Authors:** Sara Charmsaz, Kirrilee Beckett, Fiona M. Smith, Claudia Bruedigam, Andrew S. Moore, Fares Al-Ejeh, Steven W. Lane, Andrew W. Boyd

**Affiliations:** 1 QIMR Berghofer Medical Research Institute, Brisbane, Australia; 2 The University of Queensland, Brisbane, Australia; 3 Children’s Health Queensland Hospital and Health Service, Brisbane, Australia; University of Sydney, AUSTRALIA

## Abstract

Members of the Eph family of receptor tyrosine kinases and their membrane bound ephrin ligands have been shown to play critical roles in many developmental processes and more recently have been implicated in both normal and pathological processes in post-embryonic tissues. In particular, expression studies of Eph receptors and limited functional studies have demonstrated a role for the Eph/ephrin system in hematopoiesis and leukemogenesis. In particular, EphA2 was reported on hematopoietic stem cells and stromal cells. There are also reports of EphA2 expression in many different types of malignancies including leukemia, however there is a lack of knowledge in understanding the role of EphA2 in hematopoiesis and leukemogenesis. We explored the role of EphA2 in hematopoiesis by analyzing wild type and EphA2 knockout mice. Mature, differentiated cells, progenitors and hematopoietic stem cells derived from knockout and control mice were analyzed and no significant abnormality was detected. These studies showed that EphA2 does not have an obligatory role in normal hematopoiesis. Comparative studies using EphA2-negative MLL-AF9 leukemias derived from EphA2-knockout animals showed that there was no detectable functional role for EphA2 in the initiation or progression of the leukemic process. However, expression of EphA2 in leukemias initiated by MLL-AF9 suggested that this protein might be a possible therapy target in this type of leukemia. We showed that treatment with EphA2 monoclonal antibody IF7 alone had no effect on tumorigenicity and latency of the MLL-AF9 leukemias, while targeting of EphA2 using EphA2 monoclonal antibody with a radioactive payload significantly impaired the leukemic process. Altogether, these results identify EphA2 as a potential radio-therapeutic target in leukemias with MLL translocation.

## Introduction

Eph/ephrin form the largest family of receptor tyrosine kinases (RTKs) and fall into two groups based on their sequence homology, ligand specificity and structural features. Fourteen members of Eph receptors (EphA and EphB receptors) bind to eight members of ephrin ligands (ephrin-A and ephrin-B ligands) [1, 2]. In the hematopoietic system, expression of Eph/ephrin has been detected on purified populations of hematopoietic stem cells (HSCs) in both human and mouse [3–5]. Real-time quantitative PCR and flow cytometric analysis of purified HSCs in the mouse bone marrow show expression of all EphA receptors except EphA6 and EphA8, along with expression of members of ephrin-A ligand, with ephrin-A4 and ephrin-A5 being the most highly expressed [6]. Expression of Eph/ephrin has been reported in progenitor cells including erythroid progenitors, B-cells and T-cells. They have also been implicated with platelet aggregation and lymphoid development [5, 7, 8].

Members of the Eph/ephrin family are aberrantly expressed in cancer cells and tumor microenvironment where they influence tumor growth and spread [9–12]. Intriguingly, Eph receptors can have either tumor-suppressing or tumor-promoting activity depending on the cancer type [13]. In particular, increased expression of members of the Eph/ephrin system has been detected in human leukemia. EphA3 was originally identified in the LK63 pre-B acute lymphoblastic leukemia (ALL) cell line and further investigations revealed its expression in other leukemic cell lines [14, 15]. Co-expression of ephrin-B2 and EphB4 (HTK) was found in many leukemic cell lines [8]. Studies by Nakanishi et al showed up-regulation of EphA7 in ALL1-associated leukemia (ALL1/AF4 and ALL1/AF9) [16]. They have also reported expression of other EphA transcripts including EphA1, EphA2, EphA3, EphA4, and EphA6 in the MLL-AF9 and MLL-AF4 transfected K562 cells [16]. More recently, the Eph receptors have been investigated as potential targets for cancer therapy, with the most advanced therapies targeting EphA2, EphA3 and EphB4 [11]. Despite reports of Eph expression in hematopoietic cells, the role of Eph/ephrins in hematopoiesis remains to be defined.

The available literature indicates expression of EphA2 transcript at significant levels in HSCs [6] and various human malignancies however there is a limited knowledge on the specific role of this member of Eph family of RTKs in HSCs and leukemias[6]. In this report we explore the potential role of the EphA2 protein in the control of normal hematopoiesis and leukemia. To indicate the specific role for EphA2 in normal hematopoiesis, we examined hematopoiesis in EphA2 knockout mice in comparison to their wild type littermates. We have also examined the expression of EphA2 in the mouse model of leukemia and observed that MLL-AF9 induced murine leukemia have elevated EphA2 expression. EphA2 monoclonal antibody therapy has been previously used in different types of cancers that express EphA2 in this report we explored the effect of targeting EphA2 using EphA2 monoclonal antibody and radiolabeled EphA2 monoclonal antibody in leukemias initiated by MLL translocations.

## Methods

### Ethics statement

All of the human cell samples utilized for this report were collected with the donor's written informed consent in accordance with the Declaration of Helsinki, and approved by the QIMR Berghofer Medical Research Institute Human Ethics committee and Queensland Children Medical Research Institute Human Ethics committee. All documentation was stored securely as approved by the Institutional Ethics Committee.

Animal studies were conducted under approval of QIMR Berghofer Medical Research Institute Animal Ethics Committee. Mice were anesthetized with 2–3% isoflurane in oxygen and euthanized humanely by cervical dislocation or asphyxiation as approved by the Ethics Committee.

### Animals

EphA2 knockout mice were kindly supplied by Dr Naruse-Nakajima (University of Tokyo) [17] and maintained on the C57BL/6 (Jackson Laboratory, Bar Harbor, ME) background. Female mice of the congenic strains C57BL/6 (Ly 5.2) and PTPRC (Ly 5.1) were purchased from Animal Resource Centre (Perth, Australia). These mice were crossed to produce heterozygous CD45.1/45.2 mice. All the mice used in these studies were between the ages of 6–8 weeks. Mice were kept in QIMR Berghofer Medical Research Institute pathogen free animal facility according to institute protocols.

### Peripheral blood analysis and flow cytometry

After anaesthetizing the mice peripheral blood was collected using submandibular blood sampling and analyzed on a Hemavet analyzer (Drew Scientific, Oxford, CT).

Cells purified from bone marrow and spleen of mice after red blood cell lysis (BD Biosciences, San Jose, CA) were counted on a Coulter Counter (Beckman Coulter, Miami, FL). These cells were stained for mature blood cells, progenitor cells and hematopoietic stem cells [18, 19]. Cells were stained with CD3 (eBioscience, San Diego, CA 12-0031-82) for T-cells, Gr1 (Biolegend, San Diego, CA, 108412) for Granulocytes and B220 (Biolegend, 103204) for B-cells. CD71 (eBioscience, 12-0711-82) and Ter-119 (eBioscience, 17-5921-82) antibodies were used for erythroid maturation analysis.

To isolate hematopoietic stem and progenitor cells (HSPC), bone marrow cells were stained with a cocktail of biotinylated anti–mouse antibodies against antigens expressed on lineage-committed cells with a panel of biotinylated monoclonal antibodies to Ter119 (553672, BD Biosciences), CD3 (Clone 145-2C11, 553060, BD Biosciences), CD5 (Clone 53–7.3, 553019, BD Biosciences), B220 (Clone RA3-6B2, 553086, BD Biosciences), Mac-1 (Clone M1/70, 553308, BD Biosciences) and Gr-1 (Clone RB6-8C5, 553125, BD Biosciences). The lineage^+^ cells were detected with streptavidin (Biolegend, 405208) to enrich for early hematopoietic cells. These lineage^-^ cells were stained with a combination of c-kit (eBioscience, clone 2B8, 17-1171-82) and Sca-1 (eBioscience, clone D7, 25-5981-82). This population of cells contains two subpopulations known as Lineage^-^c-Kit^+^Sca-1^+^ (LKS^+^) cells and Lineage^-^c-Kit^+^Sca-1^-^ progenitor cells. Progenitor cells were stained with CD34 (eBioscience, clone RAM34, 11-0341-82), FcγRII/III (eBioscience, clone 93, 12-0161-82) to fractionate this population into granulocyte-monocyte progenitors (GMPs), common myeloid progenitors (CMPs) and megakaryocyte-erythrocyte progenitors (MEPs). LKS^+^ cells were stained with either CD34 (clone RAM34, 11-0341-82) and Flk2 (clone A2F10.1, 12-1351-82) or CD150 (clone TC15-12F12.2, Biolegend 115912) and CD48 (clone HM48-1, Biolegend 103418) antibodies to further fractionate these cells into long term-HSCs (LT-HSCs), short term-HSCs (ST-HSCs) and multipotent progenitors (MPPs).

Cell surface expression of Eph proteins were defined with 10 μg/ml of in-house anti-EphA1,-EphA2,-EphA3 and-EphA7 monoclonal antibodies or ephrinA5-Fc fusion protein and stained with SYTOX-Blue (Life Technologies, Gaithersburg, MD) dead cell marker. All cells were analyzed on an LSRFortessa flow cytometer (BD Biosciences).

### Competitive transplantation assays

Competitive transplant experiments were carried out by injecting EphA2 knockout or wild type CD45.2 donor cells together with equal numbers of double positive CD45.1/45.2 congenic competitor cells into the tail vein of irradiated (11 Gy) CD45.1 recipient mice. Blood chimerism was analyzed every 4 weeks for 16 or 24 weeks and the bone marrow and spleen chimerism were determined in the last week.

### Real-time quantitative polymerase chain reaction analysis

RNA was isolated from bone marrow using RNeasy Mini Kit (QIAGEN, Valencia, CA) and complementary DNA (cDNA) was synthesized using SuperScript III (Life Technologies) with respect to the manufacturer's instructions. Relative transcript quantification was performed with SYBR Green QPCR Mastermix (Applied Biosystems, Foster City, CA) using RotorGene 3000 Real-Time PCR system. Gene expression was calculated relative to 18S rRNA or β-actin housekeeping gene.

### Mouse model of leukemia

For primary transplantation mouse hematopoietic stem cells were obtained using fluorescence-activated cell sort (FACS) and cultured overnight on fibronectin in StemSpan media (Stem Cell Technologies, Vancouver, BC, Canada) with 100 ng/mL Stem Cell Factor (SCF, PeproTech, Rocky Hill, NJ, USA), 50 ng/mL Thrombopoietin (TPO, PeproTech.), 100 ng/mL Granulocyte colony-stimulating factor (G-CSF) and 100 U/ml Penicillin-Streptomycin (Life Technologies, Gaithersburg, MD). The cultured cells were incubated for 48 hours with supernatants of MSCV-MLL-AF9-GFP and MSCV-BCR-ABL-GFP retroviral vectors as described previously [19]. Purified hematopoietic stem cells (LKS+) were transduced with MLL-AF9-GFP or BCR-ABL-GFP containing retrovirus. Transduced green fluorescent protein-positive (GFP^+^) cells were transplanted into sublethally irradiated (5.5 Gy) recipient mice via tail vein injection. For secondary transplant 2×10^4^ GFP^+^ leukemic cells from primary transplant were injected via tail vein into sublethally irradiated (5.5 Gy) recipient mice (C57BL/6). These mice were monitored and scored for signs of leukemia every second day and mice with clinical signs of disease were sacrificed immediately [19].

To assess the potential therapeutic effect of anti-EphA2 antibody on MLL-AF9 leukemic mice, the mice were treated intraperitoneally with 0.1 mg of anti-mouse EphA2 monoclonal antibody (IF7 mAb) in PBS or vehicle only (PBS) control on alternate dates until the mice were euthanized due to progression of disease.

### Primary MLL-rearranged leukemia samples

Primary bone marrow or peripheral blood samples were obtained from patients with MLL-rearranged acute myeloid leukemia (AML, n = 3) and ALL (n = 3 pre-B ALL, 1 T-ALL), after informed consent in accordance with the Declaration of Helsinki. Mononuclear cells were recovered using Ficoll density gradient purification. The median percentage of blasts in each sample was 92.5% (range 65–96%).

### Radiolabeling and immunoreactivity

The EphA2 monoclonal antibody (IF7 mAb) and EphA3 monoclonal antibody (IIIA4 mAb) were radiolabeled with Lutetium-177 (^177^Lu, PerkinElmer, MA) as described previously [20]. The specific radioactivity was 8.1 and 8.5 mCi/mg (i.e. 300 and 316 MBq/mg) for ^177^Lu- IF7 and ^177^Lu-IIIA4 respectively. Treatments with PBS, unlabelled IF7, ^177^Lu-IF7 and ^177^Lu-IIIA4 antibodies were initiated after transplantation. The average percentage of GFP^+^ cells in mouse blood was determined using flow cytometry and the treatments were administered intravenously via tail vein injection.

### Statistical analysis

Statistical analyses were performed using GraphPad Prism Version 6.01 software. All flow cytometry data were analyzed with FlowJo software (TreeStar).

## Results

### EphA2 is dispensable for normal hematopoietic development

**Fig 1:**
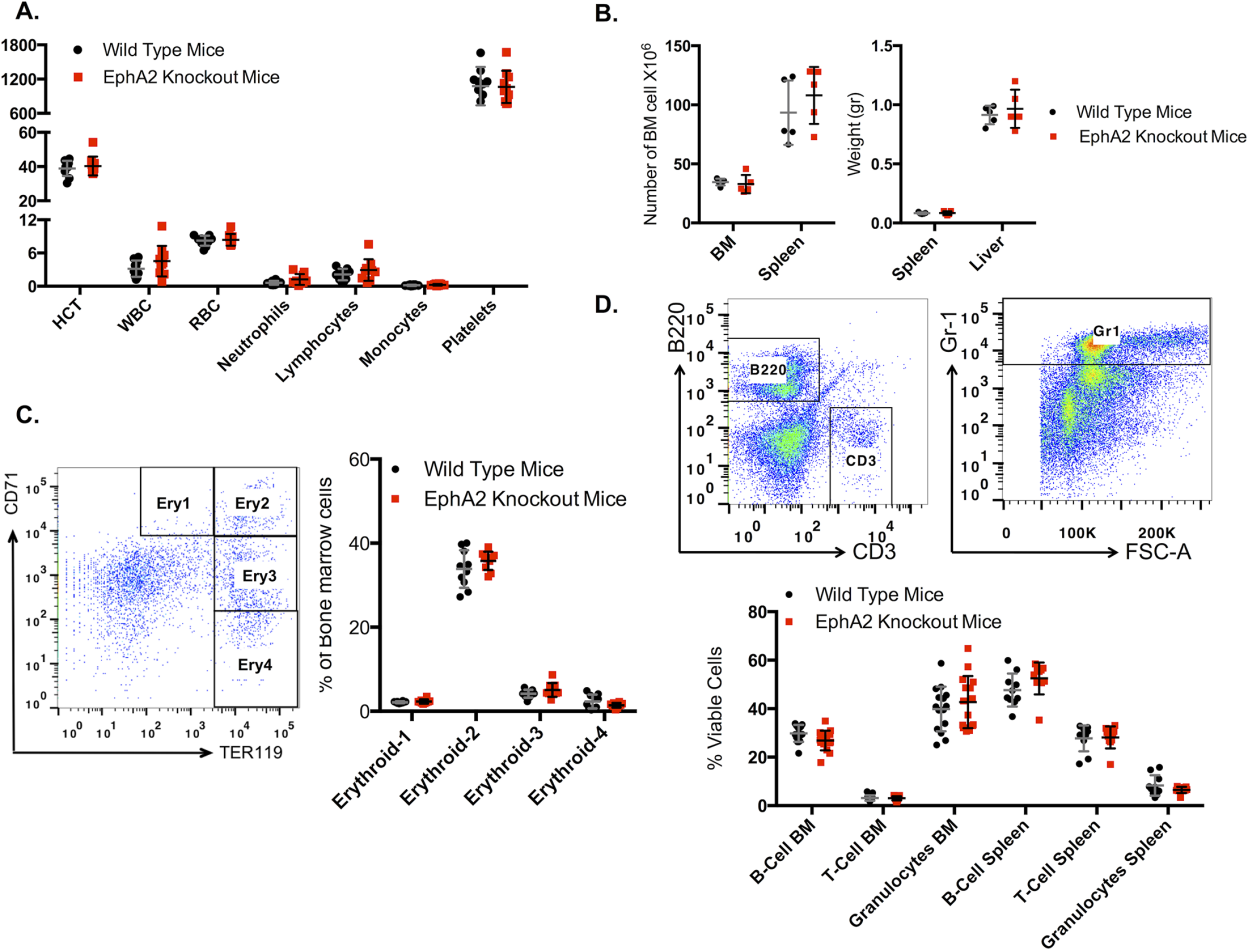
Immunophenotyping of EphA2 knockout mice compared to wild type littermates. (**A**) Full blood counts showed no significant differences in EphA2 knockout mice compared to wild type mice (n = 15, 3 independent experiments). (**B**) Number of nucleated bone marrow and spleen cells and spleen and liver weight of EphA2 knockout mice compared to the wild type littermates did not show any significant differences (n = 5). (**C**) Erythroid maturation analysis, erythroid-1 corresponding to pro-erythroblasts (CD71^high^, Ter119^mid^), erythroid-2 corresponding to basophilic erythroblasts (CD71^high^, Ter119^high^), erythroid-3 corresponding to late basophilic and polychromatophilic erythroblasts (CD71^mid^, Ter119^high^) and erythroid-4 corresponding to late basophilic and polychromatophilic erythroblasts (CD71^low^, Ter119^high^) in the bone marrow presented as percentage of viable bone marrow cells showed no differences in erythroid maturation in EphA2 knockout compared to wild type control cells (n = 15, 3 independent experiments). (**D**) B-lymphocytes (B220), T-lymphocytes (CD3) and Granulocytes (Gr-1) analysis showed no differences in B-lymphocytes, T-lymphocytes or granulocytes population in bone marrow, spleen and blood of the EphA2 knockout mice compared to the wild type control mice (n = 15, 3 independent experiments). The data represent the mean ± SEM (standard error of the mean). Unpaired *t* test was performed for statistical analyses (n) represent the number mice used in each experiment. An unpaired *t* test was performed for statistical analyses.

Blood development in mature EphA2 knockout mice, compared to normal C57BL/6 mice, showed no differences in baseline blood parameters, including hematocrit (HCT), white blood cell (WBC), red blood cell (RBC), neutrophil, lymphocyte, monocyte or platelet counts in the blood (Fig 1A). No significant differences were observed in the weight of spleen and liver in EphA2 knockout mice. The number of nucleated bone marrow and spleen cells also did not exhibit any significant differences (Fig 1B).

Analysis of the stages of erythroid maturation in EphA2 knockout bone marrow showed no perturbation of erythropoiesis when compared with wild type mice (Fig 1C). Moreover, bone marrow, spleen and blood immunophenotyping analysis indicated no significant effect from lack of EphA2 expression on particular cell lineages, including granulocyte (GR-1), B-lymphocyte (B220), or T-lymphocyte (CD-3) populations (Fig 1D).

**Fig 2:**
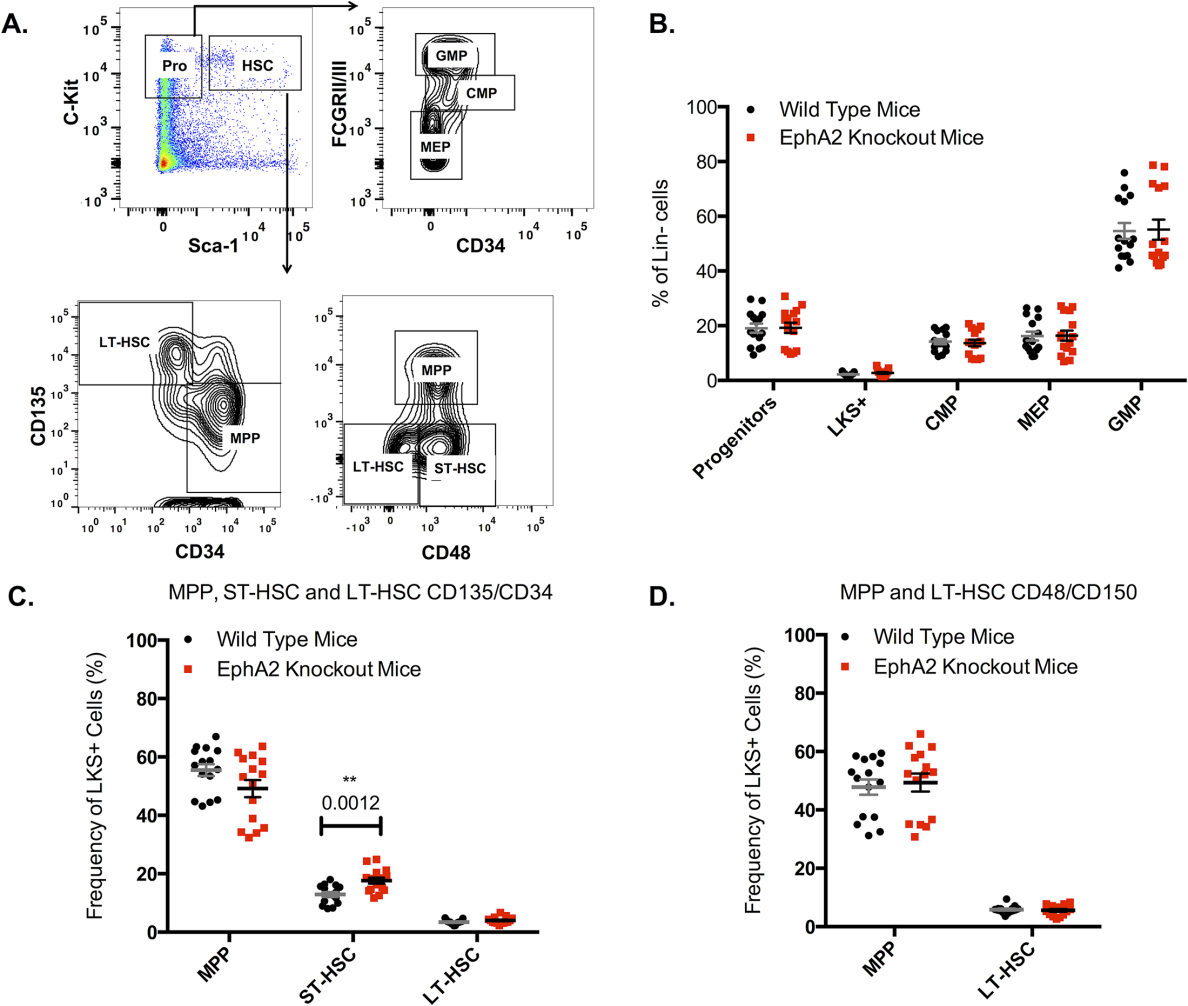
Stem/progenitor cell populations in EphA2 knockout mice compared to wild type littermates. (**A**) Gating for Progenitors (lineage^low^cKit^high^Sca-1^-^), LKS^+^ cells (lineage^low^cKit^high^Sca-1^+^ enriched for hematopoietic stem cells), CMP (lineage^low^cKit^high^Sca-1^-^CD34^+^FCGRII/III^low^), MEP (lineage^low^cKit^high^Sca-1^-^CD34^-^FCGRII/III^-^) and GMP (lineage^low^cKit^high^Sca-1^-^CD34^+^FCGRII/III^high^) presented as percentage of Lineage^low^ cells. CD34/CD135 gating for MPP (LKS^+^CD34^+^CD135^+^), ST-HSC (LKS^+^CD34^+^CD135^-^) and LT-HSC (LKS+CD34-CD135-). CD48/CD150 gating for LT-HSC (LKS^+^CD150^+^CD48^-^) and MPP (LKS^+^CD150^-^CD48^+^). (**B**) There were no significant differences in progenitors, LKS^+^, CMP, MEP or GMP population in the EphA2 knockout mice compared to wild type control mice (n = 15, 3 independent experiments). (**C**) There were no significant differences in LT-HSC and MPP population gated by CD34/CD135 antigens in the EphA2 knockout mice compared to wild type control. There were significantly more (P = 0.0012) ST-HSCs in EphA2 knockout bone marrow compared to wild type control (n = 15, 3 independent experiments). (**D**) There were no significant differences in LT-HSC and MPP population gated using CD48/CD150 antigens in EphA2 knockout mice compared to wild type control (n = 15, 3 independent experiments). Each dot corresponds to one individual mouse. The data represent mean ± SEM. Unpaired *t* test was performed for statistical analyses.

We next analyzed the numbers of immunophenotype-defined hematopoietic stem and progenitor cells (HSPCs) in the EphA2 knockout mice compared to wild type littermates (Fig 2A). This analysis showed no significant differences in the percentage of Lineage^-^c-Kit^+^Sca-1^+^ (LKS^+^), progenitors and mature myeloid progenitor cells, including common myeloid progenitors (CMPs), megakaryocyte-erythrocyte progenitors (MEPs), and granulocyte-monocyte progenitors (GMPs) (Fig 2B). To further analyze HSCs, CD34 and FLK2 (CD135) markers were utilized to isolate LT-HSCs (LKS^+^CD34^-^Flk2^-^), ST-HSCs (LKS^+^CD34^+^Flk2) or MPP, (LKS^+^CD34^+^Flk2^+^) population. A significant increase in the frequency of ST-HSCs (P = 0.0012) was observed in the EphA2 knockout bone marrow; however no significant differences in either the MPPs or the LT-HSCs were evident (Fig 2C). CD150 and CD48 markers were also used as another method to fractionate HSCs, with (LKS^+^CD150^+^CD48^-^) cell population representing LT-HSCs and (LKS^+^CD150^-^CD48^+^) representing MPPs. Similar to the previous results, there were no significant differences in the frequency of LT-HSCs or MPPs in EphA2 knockout mice compared to wild type control mice (Fig 2D). Altogether, these data suggest that EphA2 is not essential for normal blood development.

### EphA2 knockout HSCs have normal function in primary and secondary transplantation

**Fig 3:**
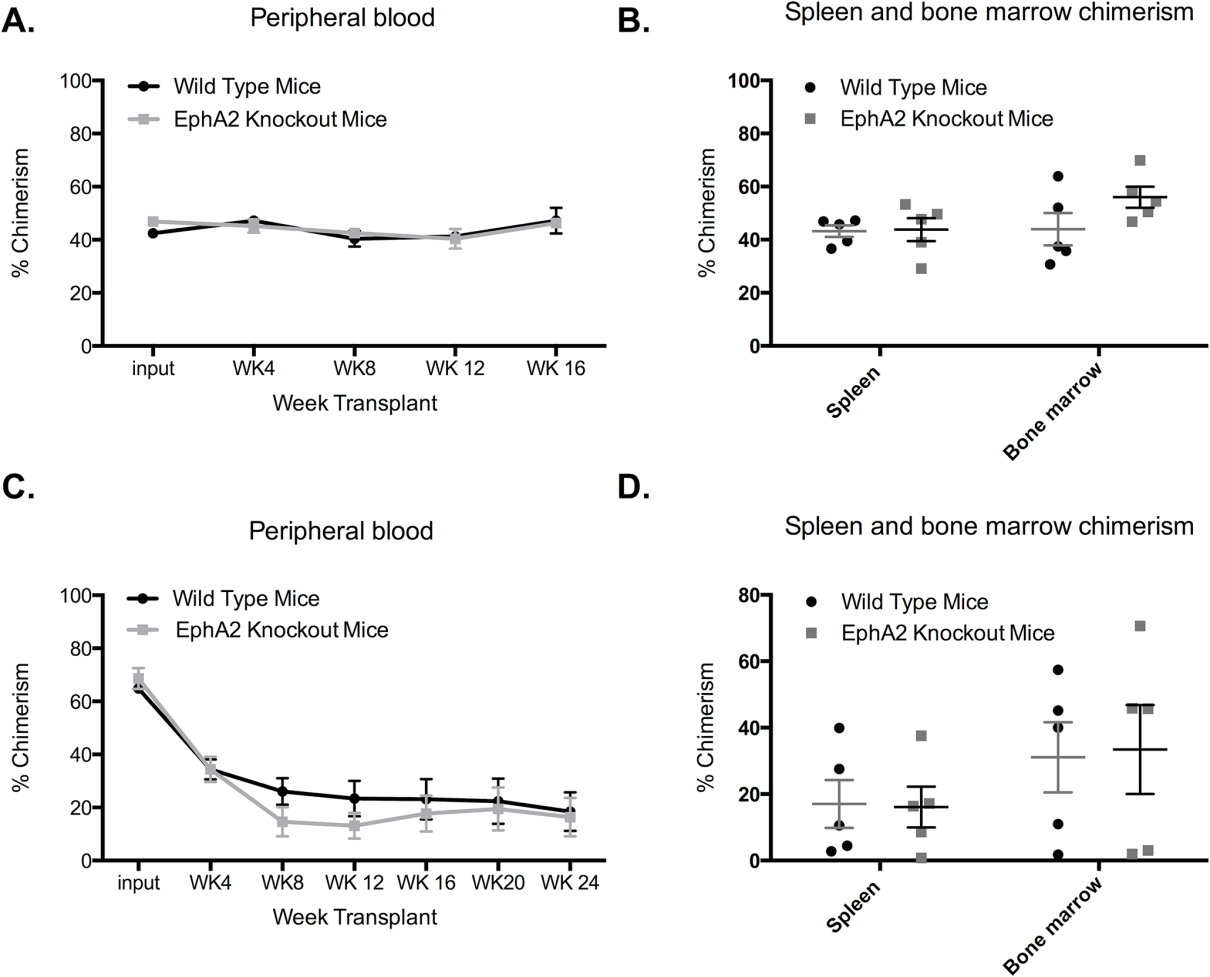
EphA2 knockout bone marrow repopulating potential in primary and secondary recipients. (A) Whole-blood chimerism at 4, 8, 12 and 16 weeks after transplantation of EphA2 knockout or wild type bone marrow cells into lethally irradiated CD45.1 recipients (n = 5). (B) Analysis of bone marrow and spleen chimerism in 16 weeks after primary transplantation. (C) Whole-blood chimerism of secondary transplant at 4, 8, 12, 16, 20 and 24 weeks after transplantation of EphA2 knockout or wild type primary bone marrow cells into lethally irradiated CD45.1 recipient (n = 5). (D) Bone marrow and spleen chimerism analysis 24 weeks after secondary transplantation. Each dot on panel A and C corresponds to mean and error from all wild type or knockout blood chimerism. Each dot on panel B and D corresponds to one individual mouse. The data presented as percentage of blood, bone marrow and spleen chimerism. The data represent mean ± SEM. Unpaired *t* test was performed for statistical analyses.

An increase in frequency of ST-HSCs in EphA2 knockout mice was observed which might indicate an altered stem cell dynamics. To verify these results, we carried out competitive bone marrow transplantation experiments using EphA2 knockout and wild type BM. No significant differences were observed between EphA2 knockout mice and congenic controls at 4, 8, 12 and 16 weeks after transplantation (Fig 3A). Bone marrow and spleen chimerism analysis also showed no statistical difference between EphA2 knockout and control mice at week 16 (Fig 3B). A secondary competitive transplantation was performed to assess whether there might be more subtle effects of EphA2 knockout on long-term stem cell self-renewal. In the secondary transplant, although some mice showed a very low percentage of chimerism, perhaps due to limiting numbers of LT-HSCs in the transplanted cell population, there were no significant differences between EphA2 knockout and control mice (Fig 3C and 3D).

### EphA2 is expressed at low levels in MLL-AF9 leukemic models

**Fig 4:**
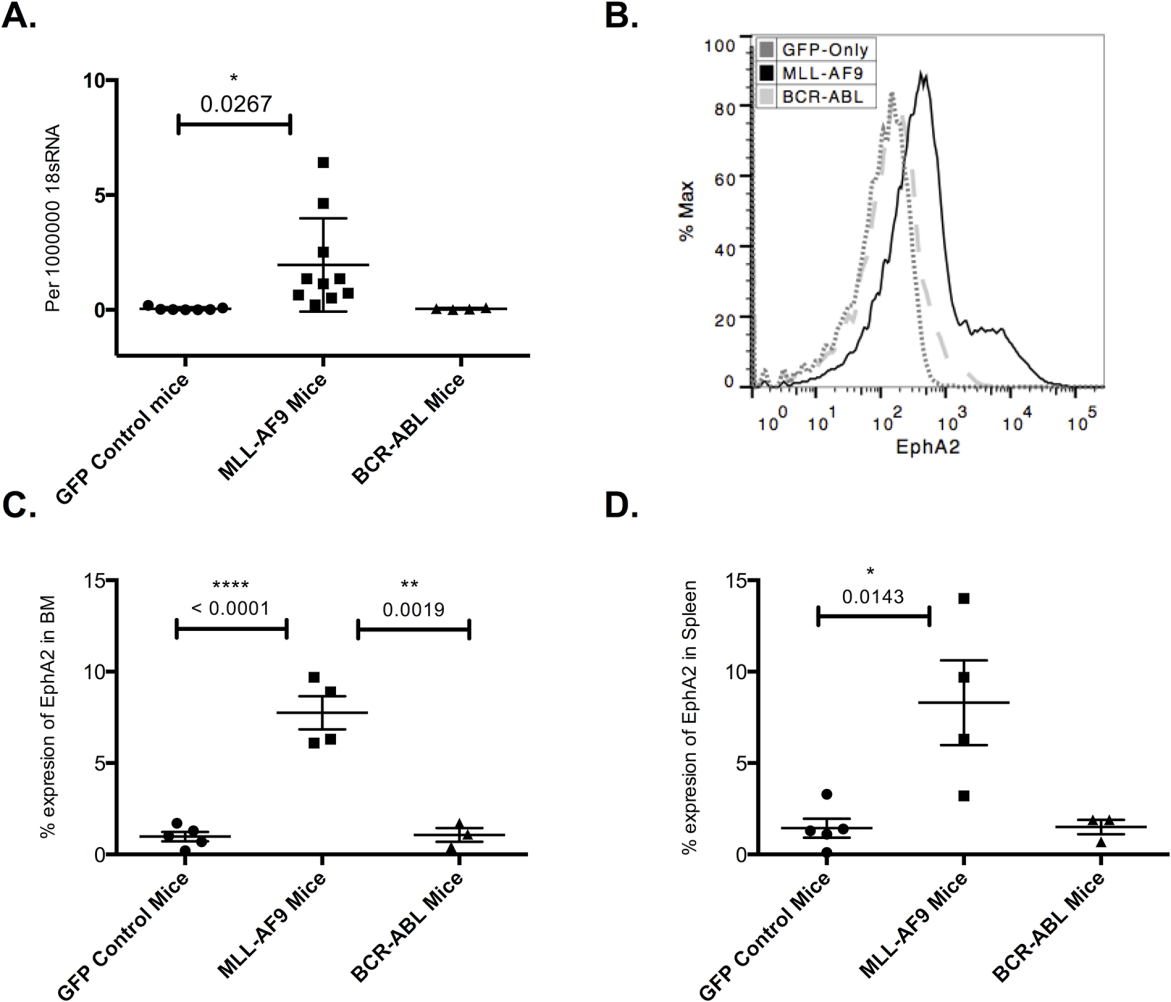
Analysis of EphA2 protein and RNA expression on GFP-control, MLL-AF9 and BCR-ABL bone marrow and spleen. (**A**) EphA2 mRNA expression levels relative to 18sRNA in bone marrow of GFP-control, MLL-AF9 and BCR-ABL mice, analyzed per 1000000 18s RNA using RT-PCR. Significantly higher EphA2 expression (P = 0.0267) in the MLL-AF9 bone marrow compared to GFP-control mice. (**B**) Representative flow cytometric overlay analysis of the EphA2 expression in GFP-control, MLL-AF9 and BCR-ABL bone marrow. (**C**) Flow cytometric analysis of the EphA2 expression in bone marrow of GFP-control, MLL-AF9 and BCR-ABL mice measured as mean fluorescent intensity, showed significantly higher EphA2 expression on MLL-AF9 bone marrow compared to GFP-control bone marrow (P <0.0001) and compared to BCR-ABL leukemic bone marrow (P = 0.0019) (n = 5 GFP-control, n = 4 MLL-AF9, n = 3 BCR-ABL, 1 biological replicates, 2 technical replicates). (**D**) Flow cytometric analysis of the EphA2 expression in spleen of GFP-control, MLL-AF9 and BCR-ABL mice measured as mean fluorescent intensity, showed significantly higher EphA2 expression in spleen of the MLL-AF9 mice compared to GFP-control mice (P = 0.0143) and higher expression compared to BCR-ABL leukemic mice (n = 5 GFP-control, n = 4 MLL-AF9, n = 3 BCR-ABL, 1 biological replicates, 2 technical replicates). Each dot corresponds to one individual mouse. The data represent the mean ± SEM. Unpaired *t* test was performed for statistical analyses.

Whilst we had shown that the expression of EphA2 is not essential for normal hematopoiesis, the increased expression of this gene in MLL transfected K562 cells and interaction of Eph tyrosine kinases with the Abl/Arg tyrosine kinase raised the possibility that EphA2 might be functionally important in MLL and/or BCR-ABL type leukemias [16, 21]. To test this, mRNA expression by RT-PCR was examined for EphA2 relative to 18S rRNA in bone marrow of GFP-control, BCR-ABL and MLL-AF9 leukemic mice. This analysis showed significantly higher EphA2 expression in bone marrow (P = 0.0267) of MLL-AF9 leukemic mice compared to GFP-control (Fig 4A). Further analysis of EphA2 expression by flow cytometry showed significantly higher EphA2 cell surface expression in the bone marrow and spleen of MLL-AF9 compared to BCR-ABL and GFP-control mice (Fig 4C and 4D).

To further explore EphA2 expression in MLL-rearranged leukemias we mined the Hemaexplorer database (http://servers.binf.ku.dk/hemaexplorer/) [22]. As is shown in supplementary S1 Fig in keeping with our analyses, EphA2 is variably but significantly over-expressed on leukemias with t(11q23) MLL rearrangement compared to normal hematopoietic stem cells, early progenitor cells (HPCs), CMPs, MEPs and GMPs (P <0.0001).

**Fig 5:**
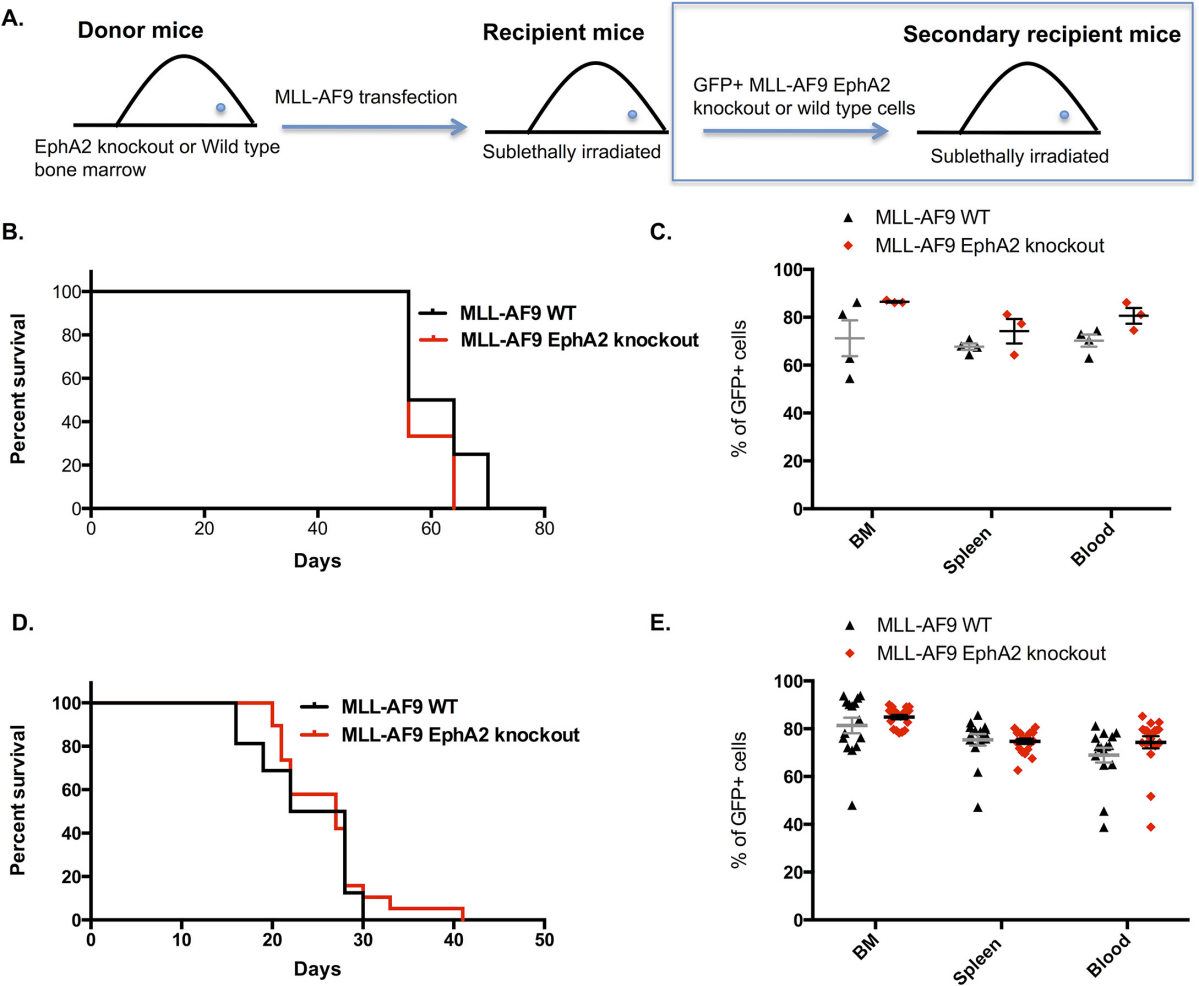
MLL-AF9 EphA2 knockout mice have similar leukemogenic potential. (**A**) Serial MLL-AF9 leukemic transplantation model. (**B**) Survival of leukemic mice transplanted with EphA2 knockout MLL-AF9–transduced hematopoietic stem cells (LKS^+^) compared to wild type MLL-AF9–transduced hematopoietic stem cells (LKS^+^) showed no significant differences between the two groups (n = 4 wild type, n = 3 EphA2 knockout, 2 independent experiments). (**C**) Percentage of GFP^+^ cells in bone marrow, spleen and blood of the EphA2 knockout and wild type MLL-AF9 bone marrow, spleen and blood at time of cull did not show any significant differences between the two groups. (**D**) Survival of secondary MLL-AF9 mice transplanted with GFP^+^ EphA2 knockout or GFP^+^ wild type MLL-AF9 cells from the primary transplant showed no significant differences between the two groups (n = 16 wild type, n = 19 EphA2 knockout, 4 independent experiments). (**E**) Percentage of GFP^+^ cells in bone marrow, spleen and blood of the secondary transplanted wild type or EphA2 knockout MLL-AF9 mice did not show any significant differences between the two groups. Each dot corresponds to one individual mouse. The data represent the mean ± SEM. Unpaired *t* test was performed for statistical analyses. The survival is presented as Kaplan-Meier survival curves (Log rank test was used for statistical analysis).

The elevated level of EphA2 expression in MLL-AF9 leukemias raised the possibility that this protein may have a role in MLL-AF9 leukemogenesis. To explore this possibility, a series of MLL-AF9 leukemic transplantation experiments were conducted (Fig 5A). Bone marrow cells from C57BL/6 wild type or EphA2 knockout were infected with the MLL-AF9 oncogene and transplanted into C57BL/6 wild type mice. The results from this transplantation showed that lack of EphA2 expression in the donor mice was dispensable for the generation of leukemia (Fig 5B and 5C).

Serial transplantations were followed up to determine if self-renewal of EphA2 knockout MLL-AF9 leukemic stem cell (LSC) was different to the wild type MLL-AF9 LSC. The GFP^+^ leukemic cells from both EphA2 knockout and wild type MLL-AF9 mice induced leukemia with similar disease latencies (Fig 5D). There were no significant differences in bone marrow, spleen and blood engraftment and in spleen and liver weight in EphA2 knockout and wild type MLL-AF9 mice at the time of cull in the secondary transplantation experiment (Fig 5E). These results indicate that absence of EphA2 does not affect self-renewal of MLL-AF9 LSCs in vivo.

### Analysis of EphA expression in human MLL-rearranged leukemia

**Fig 6:**
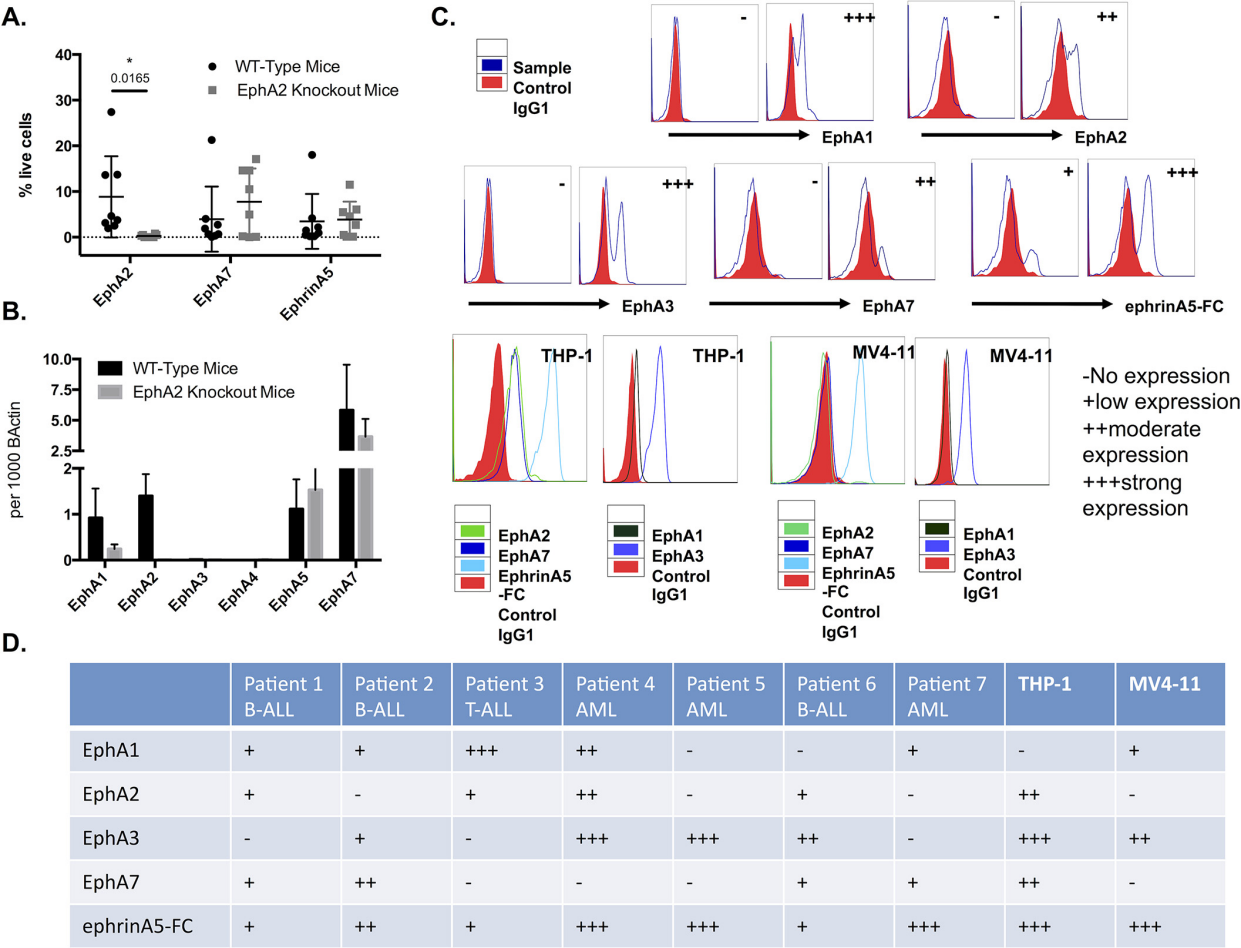
Expression analysis of EphA subfamily on mouse MLL-AF9 leukemic cells, on patient samples and human leukemic cell lines with MLL-rearrangement. (A) Flow cytometric analysis of EphA2, EphA7 and ephrinA5-Fc binding in EphA2 knockout and wild type bone marrow cells showed no EphA2 expression in the EphA2 knockout mice and significantly high expression of EphA2 in the wild type mice as expected (P = 0.0165). Comparable levels of EphA7 (P = 0.3099) and ephrinA5-Fc binding (P = 0.8710) expression were observed in EphA2 knockout and wild type leukemic cells. (B) RT-PCR analysis of EphA1, EphA2, EphA3, EphA4, EphA5 and EphA7 in EphA2 knockout and wild type bone marrow cells showed no EphA2 transcript in the EphA2 knockout mice compared to wild type MLL-AF9 (P = 0.0776) and comparable level of EphA1 (P = 0.3458), EphA5 (P = 0.7020) and EphA7 (P = 0.6167) transcript in EphA2 knockout and wild type mice. There was no expression of EphA3 and EphA4 observed in any of the MLL-AF9 mice (n = 4). (C) Representative overlay of flow cytometric analysis of EphA1, EphA2, EphA3, EphA7 and ephrinA5-Fc binding on patient samples and human cell lines. (D) Table summarizing the expression level of Eph receptors on patient samples (patients 1–7) and human leukemia cell lines (THP-1 and MV4-11). Each dot corresponds to one individual mouse. The data represent the mean ± SEM. Unpaired *t* test was performed for statistical analyses.

A possible explanation for lack of effect of EphA2 deletion in MLL-AF9 leukemia is that other Eph proteins with overlapping ephrin-binding affinities, such as EphA7 that was reported previously on MLL-AF9 leukemias [16], might show a compensatory increase in expression. To explore this possibility EphA7 and total EphA expression on EphA2 knockout and wild type MLL-AF9 leukemic cells were analyzed using flow cytometry. Total EphA expression was determined by binding of ephrinA5-Fc with high binding affinity to members of EphA subfamily. In EphA2 knockout MLL-AF9 leukemic cells, EphA7 expression was not significantly up-regulated compared to wild type MLL-AF9. However, the levels of ephrinA5-Fc binding in EphA2 knockout MLL-AF9 was not significantly different to wild type MLL-AF9 cells suggesting that, despite loss of EphA2, the complement of EphA receptors on the leukemic cells of MLL-AF9 leukemic mice was similar to control MLL-AF9 leukemic cells (Fig 6A). This supported the possibility that some compensatory increase in other Eph genes was present in the EphA2 knockout leukemic cells. To this end, we performed RT-PCR analysis of the MLL-AF9 leukemic bone marrow cells for the expression of EphA receptors in EphA2 knockout and wild type mice. This again showed no significant change in EphA7 expression in support of the flow cytometric data; there was an increase in EphA5 expression in EphA2 knockout MLL-A9 when compared to wild type MLL-AF9 leukemic cells, although this was variable and did not reach statistical significance (Fig 6B).

To further evaluate the expression of Eph family of RTKs on MLL type leukemias, seven patient clinical samples from MLL-driven leukemia and two human cell lines (THP-1 and MV4-11) bearing MLL fusion genes MLL-AF9 and MLL-AF4 were used, respectively. Expression of EphA1, EphA2, EphA3, EphA7 and total Eph, as determined by ephrinA5-Fc binding, were analyzed using flow cytometry. The results showed significant but variable expression of EphA1 and EphA3, and moderate expression of EphA2 and EphA7. Importantly, expression of total Ephs as determined by ephrinA5-Fc staining was significant on all samples (Fig 6C and 6D). Together these data showed variable level of Eph expressions on patient samples and leukemic cell lines with MLL rearrangement. In particular, EphA2 was expressed at different levels in individual leukemic samples but the significant expression in a proportion of clinical samples bearing the 11q23 chromosomal (MLL) re-arrangement, suggested that EphA2 might be a therapeutic target in human MLL-driven leukemia.

### MLL-AF9 leukemic mice treated with EphA2 monoclonal antibody immunotherapy and radio-immunotherapy treatment, delayed leukemia progression

**Fig 7:**
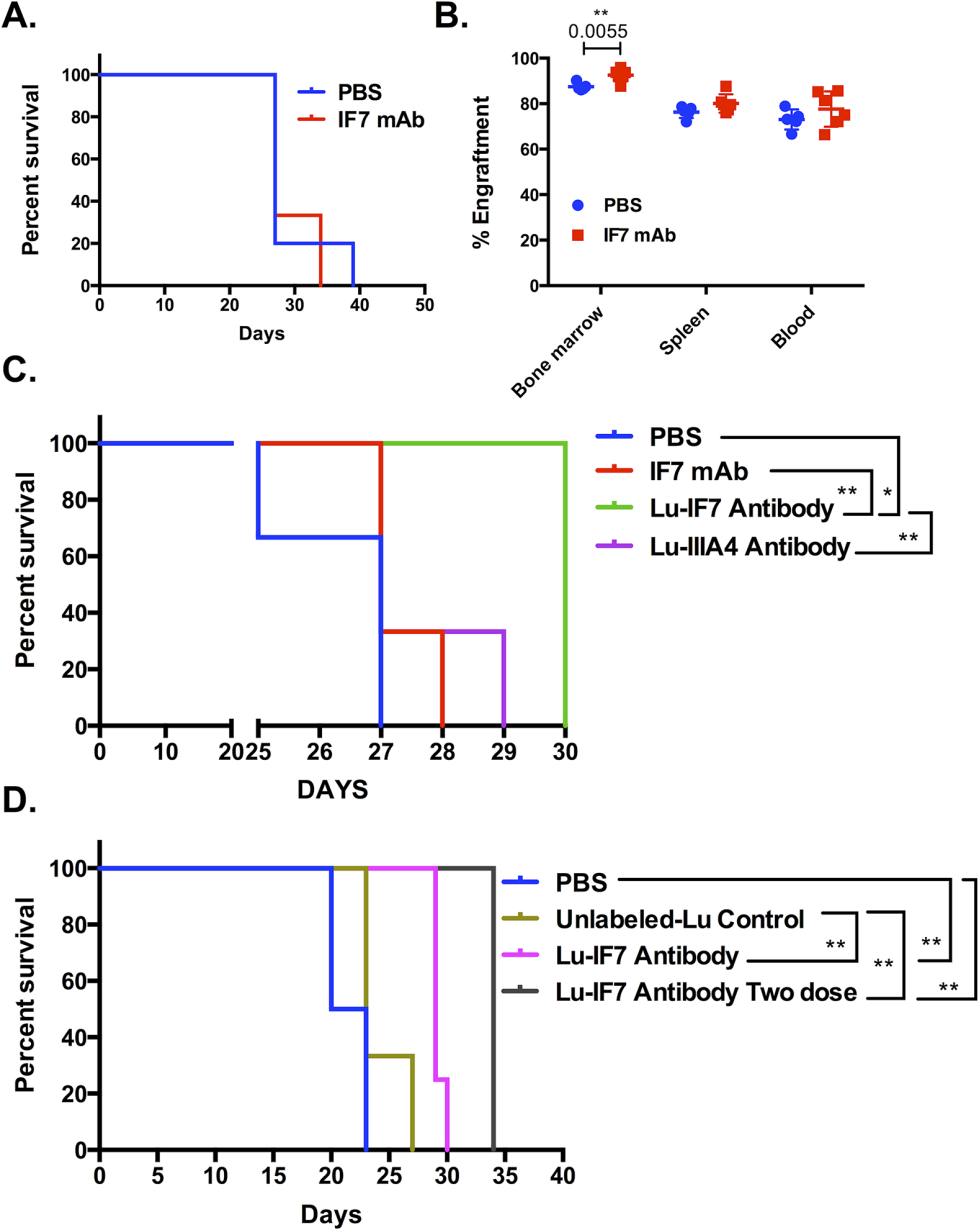
Survival and engraftment data from MLL-AF9 leukemic mice treated with PBS, EphA2 mAb (IF7), radiolabeled EphA2 (Lu-IF7) or radiolabeled EphA3 (Lu-IIIA4) antibody. (A) Survival of MLL-AF9 leukemic mice treated with IF7 mAb or PBS control showed no significant differences between the two groups. (B) Percentage of GFP^+^ cells in bone marrow, spleen and blood showed significantly higher GFP^+^ cells in bone marrow of IF7 mAb treated mice compared to PBS control (P = 0.0055). There were no significant differences observed in spleen and blood of the IF7 treated MLL-AF9 mice compared to wild type control mice at time of cull (n = 5 PBS treated, n = 6 IF7 treated). (C) Survival of MLL-AF9 leukemic mice treated with PBS, IF7 mAb, Lu-IIIA4 or Lu-IF7 antibody. While there were no significant differences between PBS, IF7 and Lu-IIIA4 antibody treated groups, a significant increase in the survival of Lu-IF7 mAb treated group was observed compared to PBS (P = 0.0140), IF7 mAb (P = 0.0100) and Lu-IIIA4 mAb (P = 0.0100) treated group (n = 3 PBS treated, n = 3 IF7 mAb treated, n = 3 Lu-IIIA4 antibody and n = 4 Lu-IF7 treated). (D) Survival of MLL-AF9 leukemic mice treated with PBS, unlabeled-Lu control and Lu-IF7 antibody showed no significant differences between PBS and unlabeled-Lu control antibody treated groups. In contrast there were significant increase in the survival of mice treated with a single dose of Lu-IF7 mAb (P = 0.0084) and more so with two doses of Lu-IF7 (P = 0.0039) compared to PBS control. The increase in survival after treatment with single dose of Lu-IF7 mAb (P = 0.0140) and two doses of Lu-IF7 (P = 0.0046) compared to unlabeled-Lu control (n = 4 PBS treated, n = 3 unlabeled-Lu, n = 4 Lu-IF7 antibody one dose and n = 5 Lu-IF7 antibody two doses, 3 biological replicates). Each dot corresponds to one individual mouse. The data represent mean ± SEM and unpaired *t* test was performed for statistical analyses. Kaplan-Meir curves were constructed in GraphPad and Log-rank (Mantel-Cox) tests were used to determine statistical differences.

Whilst EphA2 expression was not critical for the leukemic process, a significant level was detected on most cases of MLL-AF9 murine leukemia indicating that this protein might be a potential radiolabeled therapeutic target in the treatment of MLL-AF9 leukemias. To test this possibility we utilized the EphA2 monoclonal antibody (IF7 mAb) [23], which binds at nanomolar affinity to both mouse and human EphA2 and reaches a serum level > 1 mg/mL for at least 48 hours after 0.1 mg/mouse dose, a level predicted to give saturation binding of EphA2. We treated EphA2-expressing MLL-AF9 secondary transplanted leukemic mouse model with 0.1 mg of IF7 mAb or vehicle only (PBS) control every second day when the percentage of GFP^+^ cells in mouse blood reached 20–30% of viable blood cells. Mice were monitored for external (hunched back, ruffled fur, weight loss and reduced movement) and internal (white blood cell count) signs of leukemia. A clinical score based on the symptoms including WBC count (0–2), mouse weight (0–2), mouse posture and fur texture (0–2) and movement (0–2) was calculated, mice were culled once the clinical score reached 7 or more. As expected the results showed no statistical differences in survival and engraftment of leukemic mice treated with IF7 mAb compared to vehicle only PBS (Fig 7A and 7B). As we have shown previously lack of EphA2 had no effect on leukemic process and IF7 antibody treatment on its own had no significant effect on leukemogenesis, therefore we examined whether this antibody could be used to deliver a radioactive "payload" to the leukemic cells. The IF7 mAb radiolabeled with Lutetium-177 (Lu-IF7) was utilized in the treatment of MLL-AF9 leukemic mice. The mice bearing 20–30% GFP^+^ MLL-AF9 leukemia were treated with 3 MBq/21 μg of Lu-IF7 mAb, unlabelled IF7 mAb, IIIA4 anti-EphA3 mAb [14] (Lu-IIIA4) control (as MLL-AF9 leukemia do not express EphA3) or PBS control. These results showed a prolonged survival in the Lu-IF7 mAb treated group compared to PBS treated control (P = 0.0140), IF7 mAb (P = 0.0100) and Lu-IIIA4 mAb (P = 0.0100). There were no significant differences between PBS, IF7 unlabelled and the Lu-IIIA4 mAb treatment groups (Fig 7C). Additionally, we treated mice when there were 3–5% GFP^+^ MLL-AF9 leukemic cells with either one or two dose of 3 MBq/27 μg Lu-IF7. A significant improvement in the survival of mice treated with one dose and two doses of Lu-IF7 was observed. In a single dose treatment group there was a significant increase in survival of Lu-IF7 mAb compared to PBS control (P = 0.0084) and unlabeled-Lu (P = 0.0140). A greater effect was seen when mice were treated with double dose of Lu-IF7 in which there was a significant increase in survival of Lu-IF7 compared to both PBS control (P = 0.0039) and unlabelled-Lu (P = 0.0046) (Fig 7D). These data support the notion that EphA2-radioconjugate may identify and destroy AML cells in some cases of MLL-driven leukemia.

## Discussion

Expression of members of the Eph/ephrin family has been implicated with hematopoiesis and leukemogenesis. In this study we explored the role of EphA2, which has been previously implicated in hematopoiesis. EphA2 knockout mice were used to define whether EphA2 had a critical role in hematopoiesis. We showed that lack of EphA2 does not result in any significant differences in blood parameters and analysis of differentiated lineage^+^ cells showed no disruption in frequencies of these populations in the knockout mice. There were also no significant differences in the frequency of progenitors, LT-HSCs or MPPs in the EphA2 knockout mice. Although these mice exhibited a significant increase in the number of ST-HSCs further analysis of stem cell function did not reveal any significant effect on stem cell self-renewal or downstream lineage differentiation. In essence these findings indicate that these mice have a normal steady hematopoietic system. The reported expression of other members of Eph family on hematopoietic cells suggests that other members of this family of RTKs can compensate for the function in the absence of one member. For example, Ting et al. reported expression of EphA3 and EphA5, both of which are functionally similar to both EphA2 and EphA7, on hematopoietic progenitor cells, however there were no functional studies on these genes [6].

As previously described members Eph family of RTKs are expressed in many malignancies. Eph genes, including EphA2 [24, 25], have in some cases been shown to be over-expressed in cancer and to have a positive oncogenic effect [11, 26]. However, in some cases Eph genes are suppressed, in many cases by epigenetic silencing during tumor progression [27–29]. Based on reports of over-expression of EphA2 in human leukemia and our own analysis of clinical samples, we examined expression of EphA2 gene in two models of leukemia, the MLL-AF9 model of acute myeloid leukemia and the BCR-ABL model of chronic myeloid leukemia [29]. Aberrant expression of EphA2 was only significantly present in the MLL-AF9 model, as it was previously reported by Nakanishi et al. [16]. Thus, we investigated the possible functional effects of EphA2 expression on MLL-AF9 type leukemia along with the capacity of EphA2 mAb as a targeted antibody therapy or radio-immunotherapy. Our studies indicate that EphA2 expression has no significant effect on the leukemogenic process in the MLL-AF9 mouse model and lack of EphA2 did not significantly affect leukemic disease latency, severity or leukemia stem cell (LSC) self-renewal. Whilst this suggests that, as shown by others [16], the EphA2 gene is a direct transcriptional target of the MLL fusion oncogene and may not have a role in leukemogenesis. More importantly, when the total expression of Eph receptors was analyzed, the levels of expression in EphA2 knockout leukemic cells were comparable with wild type leukemic cells. This implies that there is a compensatory increase in expression of other Eph receptors, suggesting that there may indeed be a requirement for Eph function in MLL leukemia, further studies are needed, perhaps with multiple Eph knockout mice, to define the role of this family of RTKs in these leukemias.

In addition, the potential of EphA2 as a therapy target in MLL-AF9 leukemia was examined using the IF7 monoclonal antibody, which specifically binds to human and mouse EphA2 protein. There was, however, no direct anti-tumor effect delivered upon treatment of MLL-AF9 mice with EphA2 monoclonal antibody (IF7 mAb). This finding may reflect the lack of functional consequences of knocking out EphA2 in MLL-AF9 leukemic cells. Given that this antibody is mouse IgG1 isotype, it would not provide the requisite Fc function for either cellular or humoral antibody-dependent cytotoxicity, limiting its potential applicability as a naked antibody. We thus asked if we could use this high affinity and highly specific antibody to target a cytotoxic radio-active payload to the leukemic cells in an analogous approach that applied in non-Hodgkin’s lymphoma using monoclonal antibodies specific for CD20 (^131^I-tositumomab and ^90^Y-ibritumomab tiuxetan) [30]. Indeed treatment with ^177^Lutetium-labeled anti-EphA2 mAb (IF7) significantly delayed the course of MLL-AF9 leukemia particularly when two doses of the drug were administered. Given the highly aggressive nature and therapy refractoriness of this model of leukemia, this was a striking finding. More importantly, treating with ^177^Lutetium-IF7 EphA2 antibody did not result in detectable toxicity in these mice, despite concerns raised by trial of an EphA2 antibody conjugated to a drug, which was halted due to bleeding complications [31]. These studies suggest that EphA2-directed radioimmunotherapy could potentially be a new approach for targeting the EphA2 positive MLL-AF9 leukemia, particularly in combination with other anti-leukemic drugs.

In summary, we found that presence of EphA2 expression is not essential for either normal or leukemic hematopoiesis, perhaps due to functional redundancy between members of Eph family of receptor tyrosine kinases. However, targeting MLL-AF9 leukemias with radiolabeled EphA2 monoclonal antibody significantly delayed the course of the leukemia in this mouse model. This raises the possibility that targeting EphA2 might have therapeutic value in EphA2-positive leukemias.

## Supporting Information

S1 Fig EphA2 gene expression. Expression of EphA2 in AML with t(11q23)/MLL rearrangement, HSCs, HPCs, CMPs, GMPs and MEPs derived from the HemaExplorer website. (P <0.0001, 38 AML with t(11q23)/MLL, 8 HSC, 4 HPC, 3 CMP, 3 GMP and 3 MEP samples) (http://servers.binf.ku.dk/hemaexplorer/).
(TIFF)
