# Supplementary figures and images for: EphA2 Is a Therapy Target in EphA2-Positive Leukemias but Is Not Essential for Normal Hematopoiesis or Leukemia
Source: PLoS One. 2015 Jun 17;10(6):e0130692. doi: 10.1371/journal.pone.0130692 (PMC4470658; doi:10.1371/journal.pone.0130692)

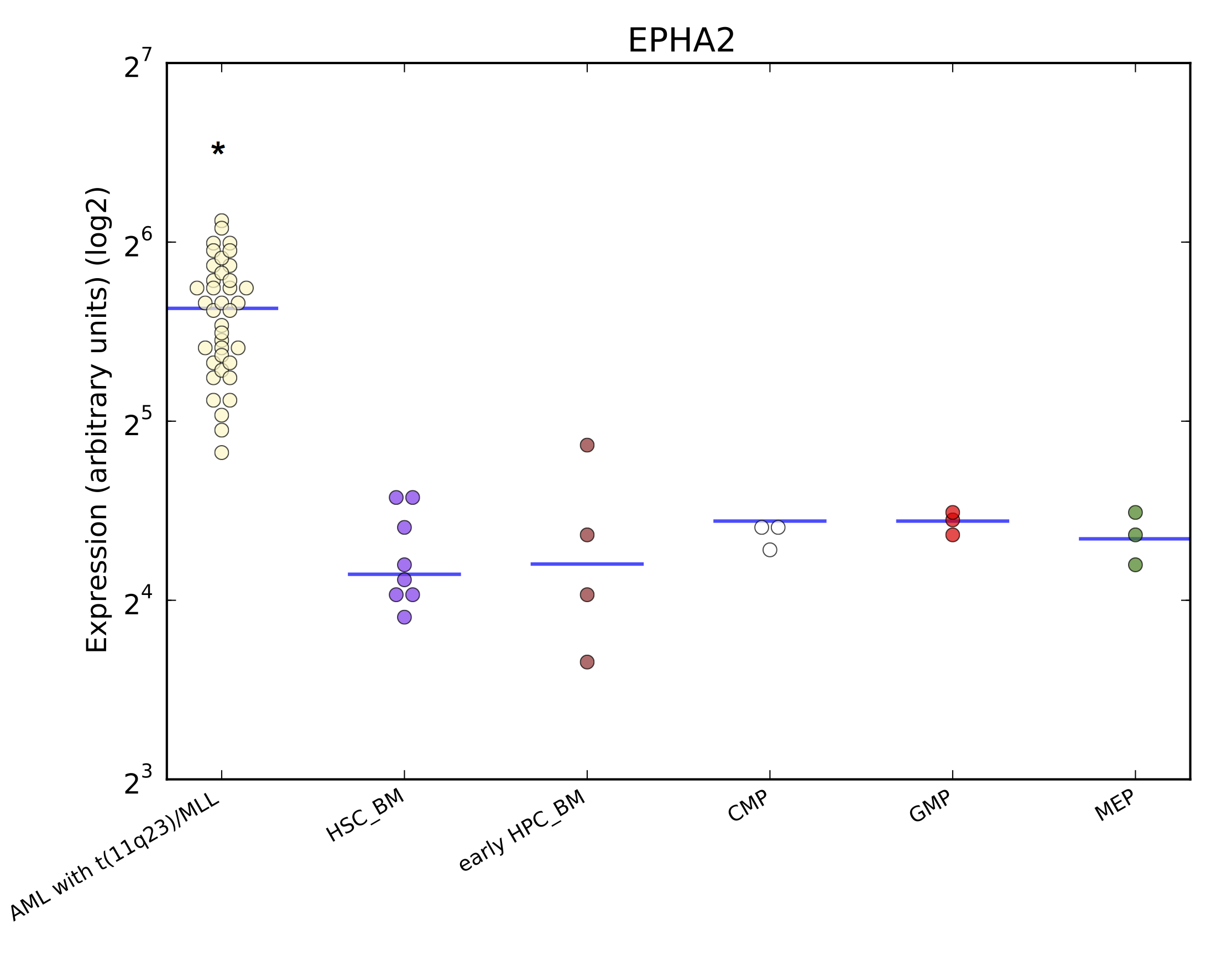

Supplement: S1 Fig — Expression of EphA2 in AML with t(11q23)/MLL rearrangement, HSCs, HPCs, CMPs, GMPs and MEPs derived from the HemaExplorer website. (P <0.0001, 38 AML with t(11q23)/MLL, 8 HSC, 4 HPC, 3 CMP, 3 GMP and 3 MEP samples) (http://servers.binf.ku.dk/hemaexplorer/). (TIFF) [file pone.0130692.s001.tiff]
